# Transperineal ultrasound is a good alternative for intra‐fraction motion monitoring for prostate stereotactic body radiotherapy

**DOI:** 10.1002/acm2.14021

**Published:** 2023-05-05

**Authors:** Bingqi Guo, Kevin Stephans, Andrew Godley, Matt Kolar, Anthony Magnelli, Rahul Tendulkar, Omar Mian, David Majkszak, Ping Xia

**Affiliations:** ^1^ Department of Radiation Oncology Taussig Cancer Center Cleveland Clinic Cleveland Ohio USA

**Keywords:** intra‐fraction monitoring, intra‐fraction prostate motion, prostate SBRT, transperineal ultrasound

## Abstract

**Purposes:**

To report our experience in a prospective study of implementing a transperineal ultrasound system to monitor intra‐fractional prostate motion for prostate stereotactic body radiotherapy (SBRT).

**Material and Methods:**

This IRB‐approved prospective study included 23 prostate SBRT patients treated between 04/2016 and 11/2019 at our institution. The prescription doses were 36.25 Gy to the Low‐Dose planning target volume (LD‐PTV) and 40 Gy to the High‐Dose PTV (HD‐PTV) in five fractions with 3 mm planning margins. The transperineal ultrasound system was successfully used in 110 of the 115 fractions. For intra‐fraction prostate motion, the real‐time prostate displacements measured by ultrasound were exported for analysis. The percentage of time prostate movement exceeded a 2 mm threshold was calculated for each fraction of all patients. *T*‐test was used for all statistical comparisons.

**Results:**

Ultrasound image quality was adequate for prostate delineation and prostate motion tracking. The setup time for each fraction under ultrasound‐guided prostate SBRT was 15.0 ± 4.9 min and the total treatment time per fraction was 31.8 ± 10.5 min. The presence of an ultrasound probe did not compromise the contouring of targets or critical structures. For intra‐fraction motion, prostate movement exceeded 2 mm tolerance in 23 of 110 fractions for 11 of 23 patients. For all fractions, the mean percentage of time when the prostate moved more than 2 mm in any direction during each fraction was 7%, ranging from 0% to 62% of a fraction.

**Conclusion:**

Ultrasound‐guided prostate SBRT is a good option for intra‐fraction motion monitoring with clinically acceptable efficiency.

## 1 INTRODUCTION

Ultra‐hypofractionated stereotactic body radiotherapy (SBRT) for prostate cancer is an emerging treatment regimen for low/intermediate‐risk prostate cancer because of its radiobiological advantage and the shortened treatment duration.^1^, ^2^, ^3^ With a reduced planning target volume (PTV) margin to spare the rectum and bladder along with a highly conformal dose distribution and a high fractional dose (7.25–8.0 Gy × 5 fractions), prostate SBRT requires high precision in each treatment delivery.^4^ Influenced by the filling status of the bladder and rectum, the position of the prostate can change both inter‐fractionally and intra‐fractionally. To account for the prostate inter‐fraction motion, KV‐CBCT is a commonly used imaging modality for each pre‐treatment setup. To reduce the intra‐fraction motion, endorectal balloons (ERB) have been used to immobilize the prostate, manually inserted in the rectum adjacent to the prostate^2^, ^5^, ^6^, ^7^, ^8^, ^9^ prior to CT simulation and prior to each fraction. The effectiveness of ERB is controversial and the use of ERB is inconvenient to patients and complicated the clinical workflow. Jones et al. reported that ERB exerted significant force on the prostate, resulting in prostate shape deformation and prostate tilt.^5^ They reported that 69% of fractions required ERB insertion adjustments to reduce the errors in the balloon positioning.^5^ Instead of immobilizing the prostate, various techniques have been developed to monitor the intra‐fraction prostate motion. For example, the Calypso system (Varian Medical Systems, Palo Alto, CA) can track the positions of implanted electro‐transponders^10^; the kilovoltage intra‐fraction monitoring (KIM) method (Varian Medical Systems, Palo Alto, CA) uses 2D radiographic imaging to detect implanted fiducial markers^11^; and ultrasound imaging can monitor the prostate displacement in real‐time.^12^

Because intra‐fractional prostate motion is a time‐dependent random walk,^13^ the question is how frequently the prostate motion should be monitored. With tracking data from 4D ultrasound, Ballhausen et al. found that variance in prostate position increased linearly over the treatment time of each fraction and fixed planning margins cannot optimally account for prostate intra‐fraction motion. Using Calypso to track prostate intra‐fraction motion, Curtis et al.^9^ found that the frequency of prostate motion monitoring should be every 60 s to detect prostate displacement >2 mm with >95% probability. From data of 89 patients receiving prostate SBRT with implanted radiofrequency transponder beacons, Lovelock reported that without continuous monitoring, intra‐fractional motion would have resulted in approximately 10% of patients not meeting the criteria of D95 of PTV >90%. In a multi‐institution study, Kupelian et al. reported that for individual patients, the number of fractions with displacements >3 mm ranged from 3% to 87%, and the number of fractions with displacements >5 mm ranged from 0% to 56%.^14^ The results of these studies demonstrated the importance of intra‐fractional prostate motion management, especially when small planning margins are applied such as seen in prostate SBRT planning. However, both Calypso and KIM methods require invasive implantation, which is a contraindication for some patients.

Transabdominal ultrasound (TA‐US) imaging was used as an imaging guidance modality (IGRT) for prostate external beam radiotherapy in the middle 90s. This IGRT method is mostly phased out clinically when KV‐CBCTs integrated with C‐arm Linacs are widely adopted. The limitations of the TA‐US included operator‐dependent, limited imaging quality, and observed discrepancy between different image modalities.^15^ Transperineal ultrasound (TP‐US, Elekta, Stockholm, Sweden) attempts to improve the image quality and reduce inter‐operator uncertainties while providing continuous intra‐fraction prostate motion monitoring. For an anthropomorpic pelvic phantom that moved with an amplitude <20 mm, Lachaine et al. reported high accuracy of the system with a mean error ≤0.2 mm ± 0.4 mm.^16^ Directly comparing implanted marker positions on 2D electronic portal images (EPI) with the TP‐US prostate motion estimate in 16 patients of 80 fractions, Grimmwood et al. reported that the two intra‐fraction detection methods were comparable.^17^ The study from Grimmwood et al. was a proof of concept and the workflow for intra‐fraction monitoring was not yet streamlined but relied on synchronization of Linac log files.

This study reports our prospective clinical trial experience of implementing Clarity Autoscan for ultrasound‐guided prostate SBRT. The initial objectives of the trial were to evaluate (a) the feasibility of ultrasound‐guided prostate SBRT and its clinical efficiency; (b) whether the ultrasound probe affects the quality of the simulation CT images, and (c) whether ultrasound‐guided SBRT improves inter‐ and intra‐ fractional prostate motion management. Since our inter‐fraction results were similar to what was observed in reference,^17^ in this study we focused on our experience of intra‐fraction motion monitoring experience.

## 2 MATERIALS AND METHODS

The IRB‐approved prospective study included 29 prostate cancer patients from 04/2016 to 11/2019, of which twenty‐three were treated with SBRT and six were treated with either hypofractionated (6 fractions) or conventional (20–35 fractions) radiotherapy. Only SBRT patients were included in this report.

### 2.1 Patient selection criteria

The patient selection criteria included: (1) patients have an established diagnosis of prostate cancer by biopsy with an intent to proceed with definitive radiation; (2) patients are ≥18 years of age and with a performance status of Karnofsky PS ≥ 70; (3) have provided their informed consent for prospective use of ultrasound for localizing and tracking prostate. Patients with uncontrolled current illness or psychiatric illness/social situations that would limit compliance with study requirements were excluded from the study. Table 1 lists patient characteristics.

**TABLE 1:** Patient and treatment characteristics.

Number of prostate SBRT patients	23
Age	46–81
Diagnosis and staging	Prostate cancer T1c‐T2b N0M0
Prescription	725 cGy × 5 Fx (LD‐PTV) 800 cGy × 5 Fx (HD‐PTV)
Distance from the ultrasound probe to isocenter	3.7–6.4 cm
Use of endorectal balloon	Endorectal balloon (19 patients, 90 fractions);No endorectal balloon (4 patients, 20 fractions)
Use of fiducial markers	With fiducial markers (6 patients, 29 fractions) No fiducial markers (17 patients, 81 fractions)
Treatment Machine	Edge machine with 6D couch (17 patients,81 fractions) Synergy‐S machine with 3D couch (6 patients, 29 fractions)

### 2.2 Clarity Autoscan ultrasound tracking system

The Clarity Autoscan system includes an ultrasound probe with the hardware and software for ultrasound image acquisition, processing, and motion tracking. The system is on a mobile cart that can be shared between the simulation and treatment rooms. Both rooms, however, need connection portals and an infrared camera installed to detect the location of the US probe. An array of four infrared‐reflective markers attached to the ultrasound probe gives real‐time feedback of the probe's position and orientation. For prostate motion monitoring, the probe is attached to a base plate that can index and lock the probe position and orientation. The ultrasound probe uses a 1‐D detector array sweeping 75‐degree angle to acquire volumetric ultrasound images. For motion monitoring, Clarity Autoscan acquires 4D ultrasound images at a frequency of 2 Hz. The patient can sense a slight vibration while the probe is sweeping. The Clarity Autoscan system is calibrated to share the same isocenter coordinates with the CT simulator and the treatment machine.

### 2.3 Clinical workflow of ultrasound guided prostate SBRT

#### 2.3.1 Simulation

**FIGURE 1:**
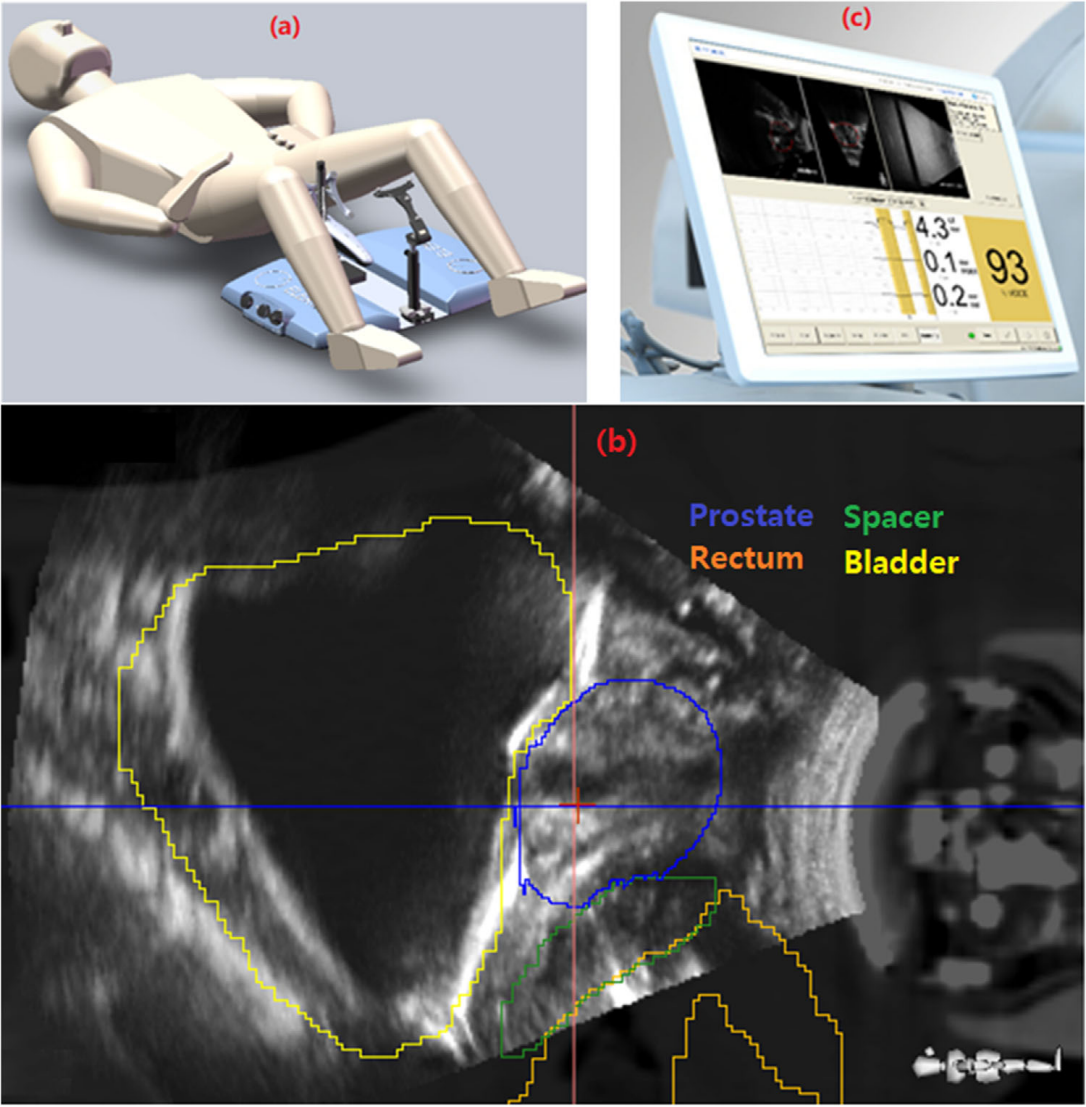
Workflow of ultrasound guided prostate SBRT. (a) Patient setup with the base plate and the ultrasound probe, (b) CT/ultrasound registration with structure contours, (c) intra‐fraction motion monitoring using Clarity Autoscan.

Patients were setup supine with legs apart and resting on a split‐knee cushion. The base plate was placed underneath the legs and indexed to the CT table. Attached to the base plate, the ultrasound probe was angled and positioned so that the probe pushed against the patient's perineum and adjusted to obtain ideal image quality. For patients with endorectal balloons, the position of the probe was adjusted to not interfere with the catheter of the balloon. Ultrasound gel was used to avoid air gaps between the probe and the skin. Figure 1a illustrates the patient setup with the base plate and ultrasound probe. A 3D ultrasound scan was acquired as the reference ultrasound. The scan protocol was chosen to ensure that the ultrasound images had a field of view encompassing the entire prostate, and the image contrast was sufficient to delineate the prostate volume. After the ultrasound acquisition, a simulation CT was acquired with the patient in the same position. The simulation CT scan followed institutional protocol for prostate SBRT with a 1.5 mm slice thickness. After the CT simulation, MRI images were acquired using a MRI simulator while patients were positioned the same as in CT simulation. In the early phase of this protocol prior to installation of the MRI simulator in our department, a Foley catheter was inserted in those patients without MRI during CT simulation to identify the urethra. A metal artifact reduction technique was applied to reduce the metal artifact caused by the ultrasound probe.

All patients were instructed to keep a full bladder and empty rectum during CT simulation and each treatment. During the three years of recruitment for this protocol, our practice had evolved from frequently using endorectal balloons, to implanting fiducial marks with/without ultrasound imaging, and to inserting SpaceOAR hydrogel (Boston Scientific, Boston, MA). Our treatment machines for SBRT were also changed from Synergy‐S (Elekta, Stockholm, Sweden) using flattened photon beams without 6D couch to Edge machine (Varian Medical Systems, Palo Alto, CA) using flattening filter free photon (FFF) beams with 6D couch. The details of patient and treatment characteristics are listed in Table 1.

### 2.4 CT/ultrasound fusion

The simulation CT and reference ultrasound images were registered based on the DICOM coordinates in the Clarity software. Manual adjustments in image registration might be made to account for the patient's movement between the time of CT scanning and ultrasound acquisition. Prostate and normal structure contours delineated on the planning CT by the physician were transferred to the ultrasound images to facilitate the manual adjustments of CT and ultrasound registration. An “ultrasound prostate” contour was delineated on the ultrasound images, which served as the reference structure for inter‐ and intra‐ fraction motion tracking during treatments. Figure 1b shows an example of CT/ultrasound registration with structure contours.

#### 2.4.1 SBRT planning

All prostate SBRT plans were created following our institutional protocol using the simulation CT plus or minus additional fused MRI imaging. The prostate and seminal vesicles (if clinically indicated) were delineated as targets as described in our previous publication.^18^ Briefly, the rectum, urethra, and bladder were delineated as organs at risk. A high dose avoidance zone (HDAZ) was created by expanding all organs at risk 3 mm in all directions. Low‐dose PTV (LD‐PTV) was created by expanding the prostate and seminal vesicles 3 mm in all directions (0 mm in posterior direction if the patient was treated with an endorectal balloon). High‐dose PTV (HD‐PTV) was created by subtracting the HDAZ from the LD‐PTV. The prescription doses were 36.25 Gy to the LD‐PTV and 40 Gy to the HD‐PTV in five fractions.

VMAT technique was used for all prostate SBRT plans, including 2−3 full arcs using the Pinnacle treatment planning system (Philips, Amsterdam, Netherlands). The first six patients were treated on a Synergy‐S linear accelerator using 6MV or 10MV flatten photon beams at a dose rate of 500 MU/min while the other 17 patients were treated on an Edge linear accelerator using 10MV FFF photon beams at a dose rate of 2400 MU/min.

#### 2.4.2 Treatment delivery

All patients were treated once every other day. Patients were setup the same way as in CT simulation. The base plate was indexed to the treatment table, and the ultrasound probe angle and position were reproduced with the guidance of the Clarity Autoscan system. A pre‐treatment kV‐CBCT was acquired for patient setup by aligning to the prostate. For the initial six patients treated on the Synergy S machine only translational corrections were applied, while for the 17 patients treated on the Edge machine 6 degrees of freedom (translation and rotation) corrections were applied using a 6D couch. A 3D ultrasound was acquired prior to KV‐CBCT. The clarity system calculates the patient shifts by registering the daily ultrasound with the reference ultrasound acquired at simulation. The ultrasound‐based shifts were not applied but recorded to compare with the shifts from KV‐CBCT.

After KV‐CBCT alignment, 4D ultrasound was reset at the treatment console to continuously monitor the prostate motion during each treatment. A 2 mm tolerance was set for the intra‐fraction motion for SBRT treatments. When the prostate motion exceeded 2 mm in any direction, the Clarity system gave an audible and visual warning to prompt therapists to manually hold the beam until the prostate moved back within 2 mm tolerance. If the prostate position displacement remained over the 2 mm threshold, repeat KV‐CBCT was utilized to reposition the patient. Figure 1c shows the Clarity Autoscan monitor during intra‐fraction motion tracking.

### 2.5 Evaluation of ultrasound guided prostate SBRT

#### 2.5.1 Efficiency

The patient setup time and treatment time were recorded for each fraction. The setup time was measured by the differences of two timestamps recorded in the Record and Verify system (Mosaiq, Elekta): the first timestamp was when the patient was brought into the treatment room, and the second timestamp was when the treatment door was closed. The imaging/delivery time was measured by the differences between the second timestamp and the time when delivery was completed. Thus, this time included our standard time‐out procedure after the treatment door closed, KV‐CBCT acquisition and image registration and review process, treatment table shifts based on KV‐CBCT imaging, and beam‐on. The total treatment time was the summation of patient setup time and imaging/delivery time.

#### 2.5.2 Prostate intra‐fraction motion

For the intra‐fraction motion, the recorded three‐dimensional prostate motions in real‐time were exported for analysis. Only intra‐fraction motion during delivery (after the KV‐CBCT alignment and before delivery finished) was included. The percentage of the time that the prostate motion exceeded the 2 mm threshold in any direction was calculated for each fraction and each patient. The number of fractions with beam holds and the number of fractions with repeated KV‐CBCT was counted.

#### 2.5.3 Statistical analysis

*T*‐test was used for all statistical comparisons. Two sample *t*‐tests were used to compare values from two different patient groups (e.g., compare the setup time between patients treated at the Synergy machine and patients treated at the Edge machine) and paired *t*‐test was used to compare values from the same patient group (e.g., compare the ultrasound‐based shifts with the KV‐CBCT‐based shifts for all patients). Table 1 lists the number of patients and treatment fractions for each group.

## 3 RESULTS

### 3.1 Feasibility of ultrasound guided prostate SBRT and its clinical efficiency

The Clarity Autoscan system was used successfully on all 23 prostate SBRT patients and 110 of the 115 treatment fractions for intra‐ fraction motion monitoring. For the five fractions in which patients were treated without ultrasound monitoring, the probe remained in the same positions as CT simulation to maintain the same anatomy. Ultrasound image quality was sufficient for visualization of the prostate and surrounding organs at risk. Figure 1b shows the ultrasound images, registered to CT for an example patient (patient #23) with prostate, bladder, rectum, and spacer contours. All structures are visible in the ultrasound image.

The patient setup time was 15.0 ± 4.9 min, the imaging/delivery time was 15.7 ± 9.0 min, and the total treatment time was 31.8 ± 10.5 min per fraction, within our scheduled time slot of 45 min for SBRT patients. Patients treated at the Edge machine had a shorter imaging/delivery time when compared with patients treated at the Synergy‐S machine (*p* < 0.01), possibly due to the use of FFF beams. The mean imaging/delivery time of all fractions was 5.4 min shorter for patients treated at the Edge machine than the group of patients treated at Synergy‐S machine. Averaged over all fractions, the use of endorectal balloons added additional 4.0 min in the setup time (*p* < 0.01).

The presence of the ultrasound probe and streaking artifacts caused by the ultrasound probe did not compromise the quality of the CT scan in the region of interest or the contouring of targets/critical structures. The artifact mostly existed in the same axial planes of the ultrasound probe, which was inferior to the targets and critical structures.

### 3.2 Intra‐fraction motion

**FIGURE 2:**
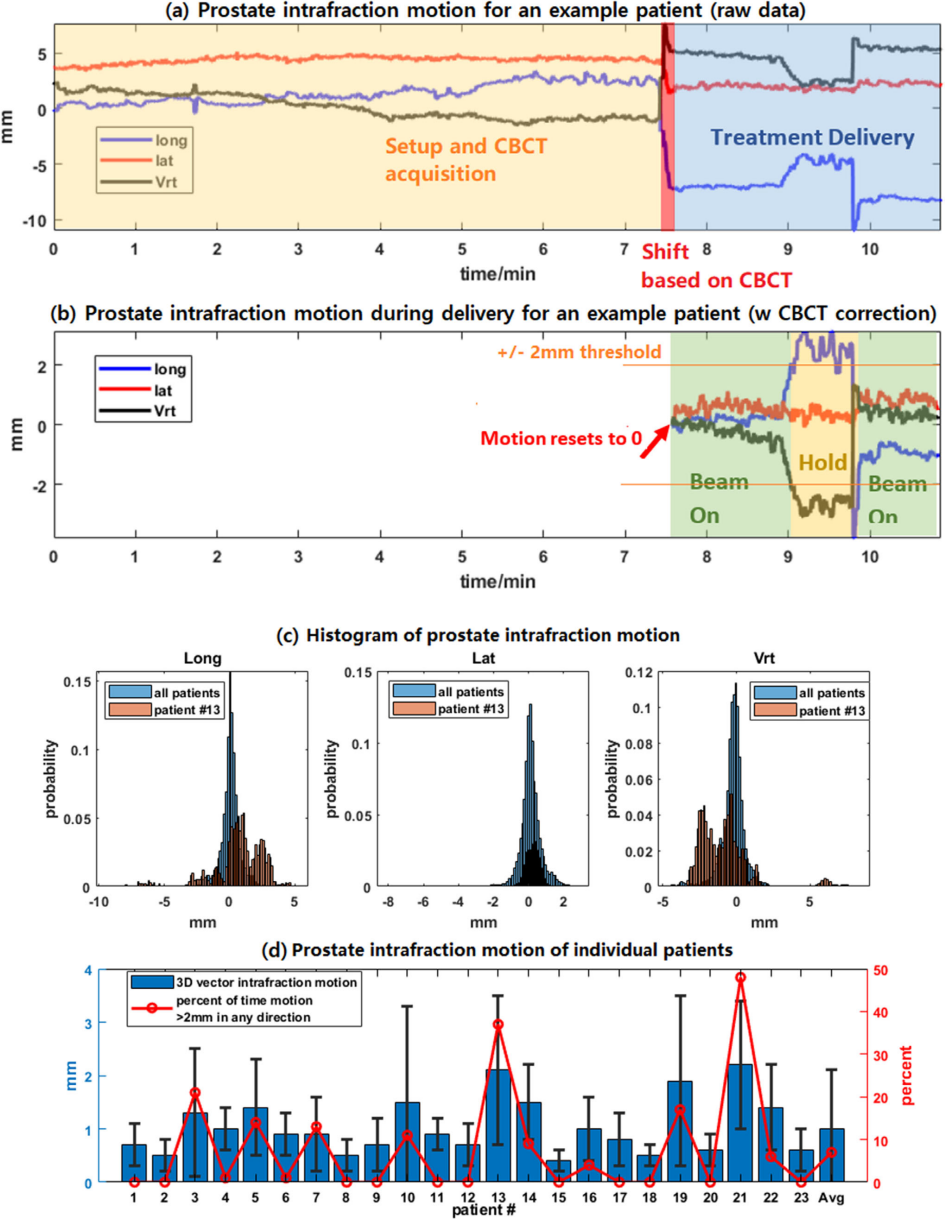
Prostate intra‐fraction motion analysis. (a) Intra‐fraction motion during treatment for example treatment fraction. + sign means shift in left, anterior and inferior directions; (b) intra‐fraction motion during beam delivery for an example treatment fraction. Motion was reset to 0 after KV‐CBCT shift correction; (c) histogram of prostate intra‐fraction motion for all patients and patient #13; (d) mean prostate intra‐fraction motion (3D vector) and percentage of time intra‐fraction motion exceeded 2 mm for each individual patient.

Prostate intra‐fraction motion was monitored during the entire treatment. However, only motion after the KV‐CBCT and before the delivery completion was analyzed, which measured 5.2 ± 2.4 min per fraction. Figure 2a,b shows the prostate intra‐fraction motion measured by ultrasound for an example fraction (patient #3). The prostate position was reset to zero after the KV‐CBCT alignment and then prostate was monitored at a frequency of 2 Hz for intra‐fractional motion. The prostate movements that were more than 2 mm in any directions triggered a warning, which prompted therapists to manually hold the beam until the prostate position returned back to within 2 mm threshold. For this treatment fraction, prostate intra‐fraction motion was out of the tolerance thus beam was hold for 50 s during delivery (25% of the delivery time). The specific cause for such a long prostate drift and then back is atypical and unknown.

Figure 2c shows the histogram of the prostate intra‐fraction motion of all patients and an example patient (patient #13). For all patients, the mean prostate intra‐fraction movements were 0.1 ± 0.6 mm in the lateral, −0.2 ± 1 mm in the vertical, and 0.1 ± 0.9 mm in the longitudinal directions. The 95th percentile of prostate intra‐fraction motion was [−1.8, 1.7] mm in the lateral, [−1.0, 1.5] mm in the vertical, and [−2.2, 1.3] mm in the longitudinal directions. For the patient who had the largest mean intra‐fraction motion (patient #13 in this study), the 95th percentile of prostate intra‐fraction motion was [−5.0 2.8] mm in vertical, [−0.8 0.9] mm in lateral and [−2.7 5.1] mm in longitudinal directions. From this cohort of patients, we found that without intra‐fraction prostate motion monitoring, an additional 5 mm planning margin to account for the intra‐fraction motion was needed.

Figure 2d shows the mean intra‐fraction motion (amplitude of 3D vector) during delivery and the percentage of time of the prostate movements exceeded 2 mm in any direction for individual patients. For all treatment fractions of all patients, the mean percentage of time when prostate moved more than 2 mm in any direction during delivery was 7%, ranging from 0% to 62% for individual fractions. For all patients, the average percentage of time when the prostate moved more than 2 mm was 8%, ranging from 0% to 48% for individual patients. In 23 of the 110 fractions (from 11 patients), the prostate moved more than 2 mm for more than 5 seconds thus a beam hold was required. Among these 11 patients, two had rectal balloons and nine without rectal balloons. In 10 fractions from five patients, additional KV‐CBCT was required to reposition the patient during treatment, one patient with the rectal balloon and four without. Because of the small number of patients without rectal balloons, statistical comparison of patients with and without rectal balloons is not reliable.

### 3.3 Difference between KV‐CBCT and TP‐US for inter‐fraction prostate alignments

Although the focus of this paper is on using TP‐US to monitor intra‐fraction prostate motion, we also compared the inter‐fraction prostate alignment between the TP‐US and KV‐CBCT. We found that the differences in shifts between the ultrasound‐based prostate alignments and KV‐CBCT‐based prostate alignments were 1.0 ± 5.4 mm in the lateral, −1.3 ± 4.4 mm in the vertical, and 0.3 ± 4.0 mm in the longitudinal directions. The differences were statistically significant in the lateral (*p* < 0.057) and vertical (*p* < 0.01) directions. In the longitudinal direction, the difference was not significant (*p* = 0.47).

**FIGURE 3:**
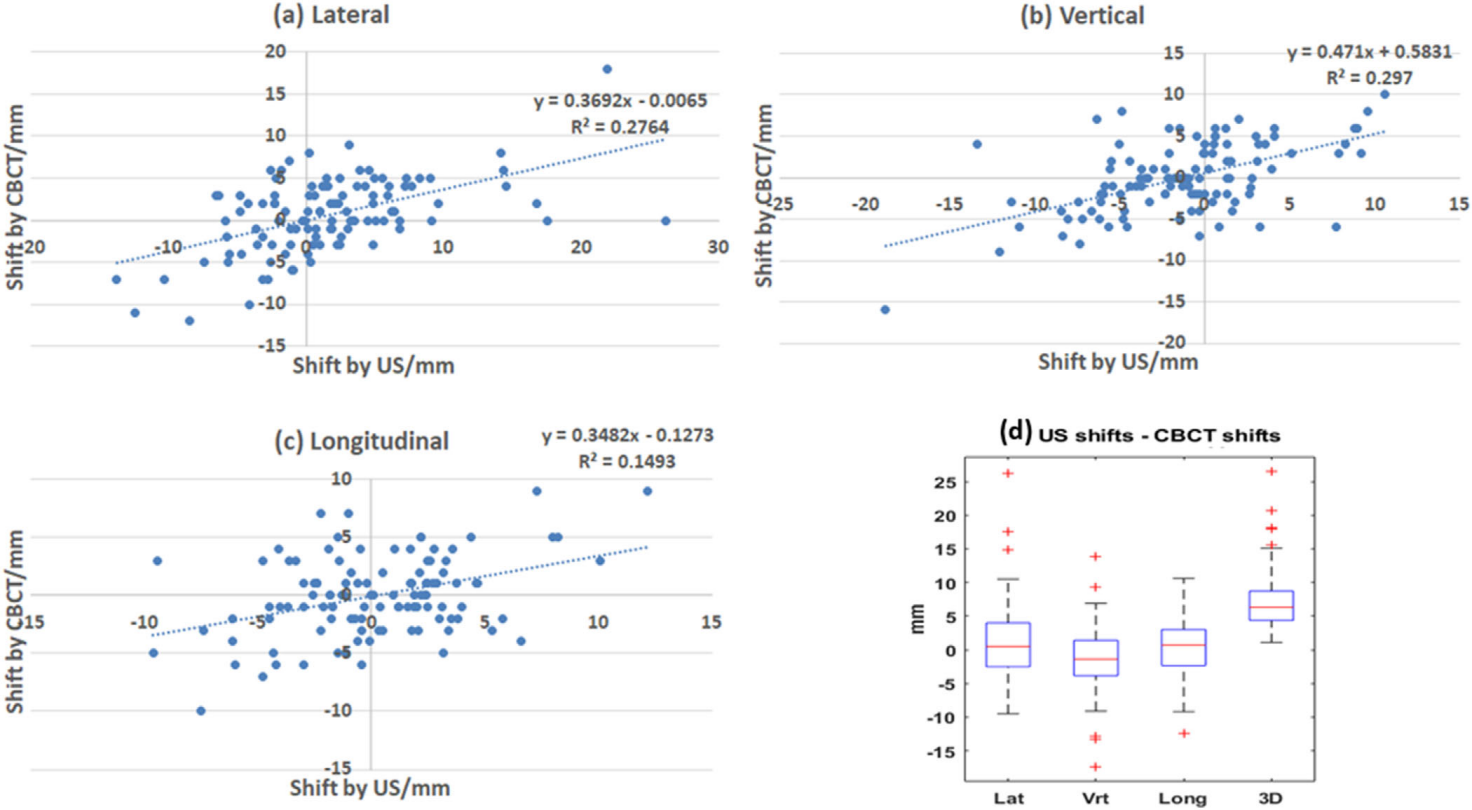
Correlation of ultrasound‐based and KV‐CBCT‐based prostate inter‐fraction motion and their differences. + sign means shift in left, anterior and inferior directions.

Figure 3 shows the relationship between the ultrasound‐based prostate alignment shifts and KV‐CBCT‐based prostate alignment shifts in the lateral, longitudinal, vertical directions, and their differences. In this study, a plus (+) sign means the prostate moved toward the left, anterior, and inferior directions and a negative (−) sign means the prostate moved toward the right, posterior, and superior directions. There were moderate correlations in lateral and vertical directions (Pearson's correlation coefficient *R* = 0.53 and 0.55, respectively) and weak correlations (*R* = 0.39) in the longitudinal direction.

## 4 DISCUSSION

In this prospective study, we presented our clinical experience of using ultrasound imaging to monitor intra‐fraction prostate motion for prostate SBRT patients. Ultrasound‐guided SBRT was feasible for all 23 prostate SBRT patients and was used in 110 of 115 fractions. Ultrasound was not used in five fractions due to technical or operational issues. Image quality from the transperineal ultrasound was sufficient to delineate the prostate, which could be used for prostate motion monitoring during treatment. The presence of the ultrasound probe did not negatively impact target and OAR delineation. Patients tolerated the procedure well. Required training for therapists is minimal and straightforward.

At our institution, ultrasound was not used for pre‐treatment target localization due to the relatively small field of view of the ultrasound images, essentially limited to the prostate and peri‐prostatic soft tissues. Daily KV‐CBCT was the imaging modality used for pre‐treatment target localization, consistent with recommendation from Grimwood et al.^17^ Data from our study and other studies^4^, ^17^ showed that using ultrasound for intra‐fraction motion management is useful, especially in the SBRT settings because of the highly conformal dose distributions, high dose gradients, and small PTV margins used for SBRT planning. Prostate intra‐fraction motion is unpredictable, varies from persistent drift to transient rapid movement, and often exceeds the typical PTV margin used in prostate SBRT planning.^13^, ^14^ Furthermore, without intra‐fraction motion monitoring, intra‐fraction margins can be patient dependent and treatment time dependent. Ballhausen et al. found that variance in prostate position increased linearly over the treatment time of each fraction and fixed planning margins cannot optimally account for prostate intra‐fraction motion.^13^ Several other methods are clinically used for monitoring intra‐fraction prostate motion, including fiducial‐based kV or MV imaging, electromagnetic‐based tracking, and in‐room magnetic resonance imaging. Except for the use of in‐room magnetic imaging, these methods require implanting fiducial markers, for which some patients cannot tolerate or have contraindications. In our practice, implanting fiducial markers is our first choice, since these implanted markers can also assist on pre‐treatment image alignment. Recently, we introduced the clinical use of a rectal spacer to separate the prostate and the rectum for patients treated with SBRT. The procedure of implanting the spacer and fiducial markers is at the same surgery session for patients who can tolerate this procedure without contraindication.

Ultrasound provides non‐invasive, real‐time, three‐dimensional intra‐fraction motion monitoring without the need for marker insertion or extra radiation to the patient. Therefore, we reserve the ultrasound method for patients who cannot tolerate marker/spacer implantation or have other contraindications. In this study, for 11 (48%) of the 23 patients and 23 (21%) of the 110 fractions, prostate intra‐fraction motion exceeded 2 mm tolerance. In some fractions, the intra‐fraction motion exceeded 5 mm and/or was out of tolerance for more than half of the treatment time. For these patients, we acquired an additional kV‐CBCT to correct for the prostate displacement. Without intra‐fraction motion monitoring, as indicated from reference,^9^ an additional margin of more than 5 mm would be needed to account for the prostate intra‐fraction motion, which is consistent with our finding in this study. In a recent phase 3 trial (PACE‐B) of comparing conventionally fractionated or moderately hypofractionated radiotherapy with five‐fraction SBRT radiotherapy, the reported acute toxicity from the five‐fraction arm was not increased.^19^ In this trial, the recommended CTV to PTV expansion was 4–5 mm isometric, except posteriorly 3–5 mm. The oncological outcome of this trial is not yet mature, but using the small posterior margin (∼3 mm) may require intra‐fraction monitoring.

Instead of monitoring the prostate motion during treatment, immobilizing the prostate is another approach, using an endorectal balloon. At our institution, this approach was a historical standard for prostate SBRT. However, the benefits of rectal balloons are controversial. While some studies showed that endorectal balloons reduced intra‐fractional prostate motion^6^ and reduced rectal toxicity,^20^ others reported that rectal balloons might push more anterior rectal wall into the high‐dose area^2^ and exert significant force on the prostate, resulting in prostate rotation and shape deformation.^21^ Our data from this study indicated that intra‐fraction prostate movement was observed among patients with rectal balloon insertions. With the introduction of SpaceOAR and triggered imaging for implanted markers to monitor prostate motion during treatment, the use of endorectal balloons becomes less common at our institution.

Besides intra‐fraction motion monitoring, TP‐US can also be used for inter‐fraction patient alignment. In comparison, for 13 patients with intact prostate treated with conventional fractionation, Fargier‐Voiron et el. reported the average shift differences between TP‐US and KV‐CBCT were −0.2 ± 2.5 mm, 2.6 ± 3.3 mm, and −0.1 ± 2.5 mm in the lateral, vertical, and longitudinal directions.^22^ In our study, we reported bigger differences (standard deviation) between TP‐US and kV‐CBCT than what Fargier‐Voiron et al. reported. A possible reason is that the majority of our patients had endorectal balloons. The balloon insertion caused rectum and prostate deformation, which resulted in larger inter‐fraction variations in prostate positions under KV‐CBCT alignments. One of the limitations of our study is that some patients enrolled for this prospective study were treated with other additional intra‐fractional management methods such as rectal balloons, rectal spacers, and implanted markers. Some patients were treated with flattened photon beams with a lower dose rate and longer treatment time per fraction. These limitations are due to the nature of a prospective study lasting three years.

## 5 CONCLUSION

Ultrasound‐guided prostate stereotactic body radiation therapy using a 4D transperineal ultrasound system was feasible with acceptable clinical efficiency and treatment plan quality. Real‐time intra‐fraction motion management can potentially improve delivery accuracy for prostate SBRT, especially when small planning margins are used.

## AUTHOR CONTRIBUTIONS

Protocol design: Ping Xia, Kevin Stephans. Data collection: Bingqi Guo, Andrew Godley, Matt Kolar, Anthony Magnelli, Kevin Stephans Rahul Tendulkar, Omar Mian, Ping Xia. Process refinements: Bingqi Guo, Andrew Godley, Matt Kolar, David Majkszak, and Ping Xia. Data analysis: Bingqi Guo. Manuscript writing: Bingqi Guo, Kevin Stephans, Ping Xia.

## CONFLICT OF INTEREST STATEMENT

Dr. Xia reports grants from Elekta, grants from NIH subcontract via John Hopkins University, during the conduct of the study; grants from Philips Medical Solution, grants from AVO, outside the submitted work. Dr. Tendulkar reports personal fees from Varian Medical Systems, outside the submitted work.

## Data Availability

Research data are stored in an institutional repository and will be shared upon request to the corresponding author.
